# 3D Microprinting of Iron Platinum Nanoparticle-Based Magnetic Mobile Microrobots

**DOI:** 10.1002/aisy.202000204

**Published:** 2020-11-13

**Authors:** Joshua Giltinan, Varun Sridhar, Ugur Bozuyuk, Devin Sheehan, Metin Sitti

**Affiliations:** Physical Intelligence Department, Max Planck Institute for Intelligent Systems, Stuttgart 70569, Germany; Physical Intelligence Department, Max Planck Institute for Intelligent Systems, Stuttgart 70569, Germany; Physical Intelligence Department, Max Planck Institute for Intelligent Systems, Stuttgart 70569, Germany; Physical Intelligence Department, Max Planck Institute for Intelligent Systems, Stuttgart 70569, Germany; Physical Intelligence Department, Max Planck Institute for Intelligent Systems, Stuttgart 70569, Germany, School of Medicine and School of Engineering, Ko$ University, Istanbul 34450, Turkey, Institute for Biomedical Engineering, ETH Zurich, Zurich 8092, Switzerland

**Keywords:** 3D microprinting, biocompatible FePt nanoparticles, magnetic microrobots, two-photon polymerization

## Abstract

Wireless magnetic microrobots are envisioned to revolutionize minimally invasive medicine. While many promising medical magnetic microrobots are proposed, the ones using hard magnetic materials are not mostly biocompatible, and the ones using biocompatible soft magnetic nanoparticles are magnetically very weak and, therefore, difficult to actuate. Thus, biocompatible hard magnetic micro/nanomaterials are essential toward easy-to-actuate and clinically viable 3D medical microrobots. To fill such crucial gap, this study proposes ferromagnetic and biocompatible iron platinum (FePt) nanoparticle-based 3D microprinting of microrobots using the two-photon polymerization technique. A modified one-pot synthesis method is presented for producing FePt nanoparticles in large volumes and 3D printing of helical microswimmers made from biocompatible trimethy- lolpropane ethoxylate triacrylate (PETA) polymer with embedded FePt nanoparticles. The 30 μm long helical magnetic microswimmers are able to swim at speeds of over five body lengths per second at 200 Hz, making them the fastest helical swimmer in the tens of micrometer length scale at the corresponding low- magnitude actuation fields of 5-10 mT. It is also experimentally in vitro verified that the synthesized FePt nanoparticles are biocompatible. Thus, such 3D-printed microrobots are biocompatible and easy to actuate toward creating clinically viable future medical microrobots.

Mobile microrobots, sub-millimeter scale machines with wireless mobility, are envisioned to revolutionize healthcare through minimally invasive medicine by accessing the regions of the body that are difficult or not possible to reach by conventional tools.[^1^] Magnetic materials are a popular choice to enable mobility due their ability to operate at centimeter-scale distances from the magnetic field source and safely in biological systems.[^1c,2^] Most studies of microrobots have utilized cobalt, nickel, or neodymium iron boron (NdFeB) as ferromagnetic materials.[^3^] These materials have enabled investigation into various actuation and control methodologies with lower magnetic fields and gradient magnitudes.[^1f,4,5^] However, they are toxic and do not exhibit their desired magnetic properties with particle sizes smaller than 1 gm, due to the presence of only a single magnetic domain. Thus, the majority of in vivo and in vitro tests have focused on the use of commercially available biocompatible superparamagnetic iron oxide nanoparticles (SPIONs).[^6^] However, SPIONs are magnetically very weak and not ferromagnetic, which cannot provide high-force/torque actuation in low magnetic fields.

The 3D additive manufacturing process is able to provide more flexible design rules than conventional subtractive manufacturing, which has limited capabilities at the single-micrometer scale, and 2D photolithography-based techniques, which involves the buildup of multiple layers. NdFeB microparticles can be infused into extrusion-based additive machines and magnetized in a preferred direction immediately prior to 3D printing.[^7^] Recent works in two-photon lithography have enabled the fabrication of almost any 3D configuration with a resolution as low as 200 nm in the *xy-axis* and 500 nm in the *z-axis*.[^8^] Different functional coatings can be applied to these structures through selective polymerization in various precursor materials. This method has been used to attach catalyst metal (Pt) nanoparticles onto the interior of a micromotor to provide propulsion.[^8d^] Metal nanoparticles can be introduced into liquid pre-polymer solution and can be trapped in the polymer matrix to functionalize the resulting structures. SPIONs are used to create biocompatible and biodegradable hydrogel, which are 25 gm long helices that could swim with speeds up to 10 gm s^-1^.[^9^] Two-photon lithography can also be used to make complex designs for electroplating templates. While this provides a method to produce 100% metal- based magnetic 3D structures, the design process is considerably complex due to the variable electroplating speeds and escape of gaseous by-products.[^10^] Another method to achieve all-metal magnetic structures using two-photon lithography is to use hybrid organic-inorganic precursors to embed metal ions in the printed structure. After pyrolization, the organic material is burned off, and only a metallic structure remains, though while retaining the original shape, the structure shrinks by a factor of 5.[^11^]

For biocompatible magnetic microrobots toward medical use, fully metallic structures may not be needed if the net magnetization is sufficient for the desired function. Also, the polymer matrix can be used to incorporate therapeutic, medical imaging, and other functional compounds.[^12-15^] Since the early 2000s, iron platinum (FePt) alloys have been of interest to researchers of magnetic storage media due to their high remanent magnetization and coercivity.[^16-18^] These films have been deposited through sputter deposition, though the best results have been obtained through the annealing of self-assembled monolayers. To obtain these monolayers, highly controlled experimental conditions are required, and a small volume is produced. Thus, these methods have been inaccessible to researchers outside of the magnetic material synthesis field. Nanoparticles that have weaker magnetic properties have been used as magnetic resonance imaging (MRI) contrast materials and for potential biocompatible hyperthermia techniques.[^19, 20^]

Recently, Kadiri et al. have used physical deposition to deposit a 350 nm section of highly ordered FePt to enable the actuation of biocompatible helical microswimmers.[^21^] While this form of FePt possesses greater intrinsic properties than the method presented here, such process cannot produce 3D-printable complex microrobots, which are essential for different specific applications. Therefore, we present the synthesis and characterization of FePt nanoparticles for two-photon lithography-based 3D microprinting of biocompatible and ferromagnetic microrobots. We 3D print biocompatible helical microrobot structures made of trimethylolpropane ethoxylate triacrylate (PETA; 912 Da molecular weight) with embedded FePt nanoparticles. Such FePt/PETA helical microswimmers have swimming speeds 500% higher than equivalently produced microrobots with embedded SPIONs. We also show that the in vitro cytotoxicity of untreated FePt nanoparticles is negligible after 24 h, though cell division is inhibited at some level.

The FePt nanoparticle synthesis process is shown in Figure 1A. A single-round bottom flask is used with the mouth of the flask connected to the condenser column top to ensure Argon, heavier than air, can purge the system of residual atmosphere. A vacuum pump is used in aiding the purging, and a pressure sensor is used to monitor the system. Synthesis details are provided in the Experimental Section. Magnetic characterization is conducted by a vibrating sample magnetometer (VSM) (Microsense EZ7). The particles were weighed and affixed to a thin wax layer, which, in turn, is mounted to the magnetometer’s sample holder. The hysteresis loop on Figure 1B shows that at the maximum field of 1.8 T, an intrinsic magnetization maximum of 60.3 emug^-1^ is achieved. The remanent magnetization is approximately half the saturation value, 32 emu g^-1^. The coercive field is slightly above 300 mT, placing the coercive field well above the actuating fields of electromagnetic coil systems, apart from MRI scanners, but an order of magnitude greater than strong elemental permanent magnetic materials, such as cobalt and nickel.

X-ray diffraction (XRD) is used to ensure that the synthesized FePt nanoparticles have the desired crystallography and can help give an approximation of the elemental composition of the particles. This is useful for engineering particles to have higher coercive fields, which occur when the particles are comprised of a ratio of 55:45 iron to platinum along with an annealing temperature of 600°C.[^16a^] The peaks are fitted using a Bruker XRD software (PANalytical HighScore Rel.: 3.0), and the spectrum shows characteristic peaks at (001), (110), (111), (200), (210), (112), and (200). These compare with the characteristic peaks of the FePt nanoparticles demonstrated in the literature.[^16a,b^] The peaks are indicative that the crystallinity of the particle becomes more highly ordered, from face-centered cubic (FCC) to face-centered tetragonal (FCT), which is required to obtain the desired strong magnetic properties.[^16a,b,22^] In addition, the sharpness of the peaks increases with the particle size when the particle diameter is on the single-nanometer scale.[^23^]

A particle crystal structure is observed by transmission electron microscopy (TEM). Figure 1D-G shows the TEM images of the representative nanoparticles. Such images show the presence of nanoparticles in the range of 2-50 nm. The particles are polydispersed due to the nature of the colloidal synthesis process. Figure 1E shows the magnified image of a single nanoparticle, where the particle is highly crystalline in nature, and its crystal facets can be observed. The presence of crystalline structures increases the magnetic properties of the FePt nanoparticles and makes them more viable for microrobotic applications. Electron energy loss spectroscopy (EELS) provides elemental information and the distribution of Fe and Pt composition within a nanoparticle. Figure 1F,G shows the EELS images of FePt nanoparticles and their colorized image, with Fe in green and Pt in red. From Figure 1G, it is shown that the Fe forms in the particle outer shell, and the Pt is mostly present inside the particles. The FePt alloy is rather homogeneous within the particle, whereas the Fe part is segregated toward the outer shell. This is possibly a function of the annealing process length, phase separation, and subsequent oxidation of Fe.[^23^] The presence of Fe on the outer shell has the potential to make the particles more biocompatible. Further energy-dispersive X-ray spectroscopy (EDS) measurements inside TEM are obtained to validate the presence of only Fe and Pt within the nanoparticle and also their elemental composition. EDS images in Figure S1, Supporting Information, show that the elemental composition is 58% Fe and 42% Pt, indicating that the synthesized FePt nanoparticles are similar to those present in the literature.[^16a^] A dynamic light scattering (DLS) analysis of the nanoparticles is also shown in Figure S2, Supporting Information. They show the hydrodynamic radius of the nanoparticles being around 72 nm. The hydrodynamic radius is always larger than the actual radius of the particles, indicating that all the particles are smaller and there is no or negligible aggregation.

Pre-polymer solution is prepared for two-photon lithography by adding 500 mg Irgacure 369, to 15 mL of PETA and mixing well. Magnetic helices are fabricated by dispersing the particles in polymer solution to achieve the desired concentration. Approximately 1 mL of particle-polymer solution is generated at a given time, which could be used for three printing sessions. Vortexing of the Eppendorf tube is performed for 2 min before placing the solution on the glass slide. Shaking is not used to prevent the formation of gas bubbles in the solution. A spatula is used to transfer the resist to the glass slide before insertion into the two-photon lithography system (Nanoscribe GmbH, Photonic Professional GT). To ensure proper 3D printing, the interface of the glass slide and solution is found at the center, and this value is not deviated. The same method of loading and printing can be translated to other resins, such as IP-L 780 (Nanoscribe). We observe the same structure and loading of the FePt nanoparticles in such new resin case.

Scanning electron microscopy (SEM) with energy-dispersive X-ray (EDX) spectroscopy is performed to confirm the 3D-printed helices and their composition. Figure 2 A shows an illustration of a single helix with nanoparticles embedded throughout its structure. It has a diameter of 3 μm, a center-to-center length of 30 μm, and three helical turns. This design is used in all experiments in this article and in the Supporting Information. Figure 2B shows a single representative helix SEM image. Figure 2C shows the EDX spectrum of the Pt signal, used to analyze the distribution of particles throughout the structure. While the signal-to-noise ratio is lower than Figure 2D, the signal of the carbon component of PETA, this confirms a relatively uniform distribution of Pt throughout the printed helix.

Swimming speed characterization experiments were performed under an inverted optical microscope (Zeiss Axio Observer A1, Carl Zeiss) fitted with a custom five-electromagnet coil system described by Alapan et al.[^24^] Figure 3 A shows the concept illustration of a 3D-printed PETA microswimmer with embedded FePt nanoparticles with forward and rolling velocity components. Figure 3C shows the swimming speed of the helix printed with 5mgmL^_1^ density of SPIONs [^15a^] and the synthesized FePt nanoparticles. The microswimmers were tested in a rotating magnetic field of 5 mT magnitude. A maximum speed increase from 10 to 50 pms^-1^ for the FePt nanoparticle- embedded helical microswimmers is observed, which is faster by a factor of 5. A subsequent batch of microswimmers using 10mgmL^_1^ density of FePt nanoparticles is tested, and the results are shown in Figure 3B. This sample is tested at 5 and 10 mT rotating field magnitudes. As SPIONs were not able to be loaded at 10mgmL^-1^ due to 3D-printing limitations,[^8a^]another common magnetic material for microrobots, nickel, is chosen and sputter-coated onto microswimmers at 100 nm thickness, the maximum amount of nickel typically demonstrated in the literature.[^25^] While nickel resists oxidation, its toxicity typically requires a barrier nanofilm layer, such as gold, to prevent undesired destruction of biological tissues.

The FePt swimmer is able to reach the speed of ≈175 pm s^-1^, over five body lengths per second, at 200 Hz in a 10 mT rotating magnetic field. This is around 25% increase in speed and 35% increase in step-out frequency over the microswimmer with 100 nm thick sputtered nickel. Video S1 and S2, Supporting Information, provide examples of a helical microswimmer swimming out-of-plane, away from the surface, and in a square path in-plane; Figure 3D provides the snapshots of the square pathfollowing video. The speed of the microswimmer in the linear regime, from 0 Hz to the frequency which yields the peak speed, is dependent on the spiral’s geometry. The fact that the samples in Figure 3B and the samples in Figure 3C are collinear shows that the magnetic materials are not affecting the geometry of the spiral. Principally, if the particles were in large aggregates, then surface roughness could be altered and change the linear velocity-frequency response. This reaffirms the EDX observation in Figure 2. Table 1 shows the properties of the common magnetic materials as particles or films used in the fabrication of magnetic microrobots.

As these magnetic microrobots are envisioned to be utilized inside the human body [^a,b, 2^] for potential medical applications, it is advantageous that the FePt microswimmers are biocompatible, though previous studies [^8b^] have shown that SPIONs- embedded PETA structures are completely biocompatible. This is due to the embedding of the nanoparticles within the matrix of the PETA, thus effectively shielding them from any form of interactions with the cells. The PETA microswimmer with embedded FePt can be removed from the targeted region after they deliver their cargo. While previous studies have shown that some synthesized FePt nanoparticles are biocompatible depending on the synthesis conditions, [^19, 20b, 21^] in vitro biocompatibility of the synthesized FePt nanoparticles with the given specific synthesis process details in this study is tested at various concentrations up to 500 μgmL^-1^ on Murine macrophage cells (J774A). The cytotoxicity of the particles is measured by incubating the particles along with the cells for two different time periods: 1 and 24 h. Live/dead staining of the cells is performed to measure their viability and their viability at various concentrations of FePt nanoparticles at two time points. The fluorescent images of the viable cells in green are shown in Figure 4B, and the percentage viability is shown in Figure 4A. Though the particles are viable, a reduction in the number of cells after 24 h is observed, indicating that the particles affect the cell growth. The particles can be made more viable using a biocompatible surface coating, which can also be functionalized, as a future work.

In this article, we have shown a 3D-microprinting process to manufacture biocompatible 3D magnetic microrobots using two-photon lithography and embedded strong ferromagnetic FePt nanoparticles. Helical microswimmers prototyped with this process have shown to be five times faster than those with SPIONs. Thus, this is a key milestone step toward real-word medical applications of biocompatible magnetic microrobots by producing faster and easier-to-actuate microrobots in the tens of micrometer length regime, which is important for ensuring control in biological environments.[^3d^] In addition, both the structural and nanoparticle components of these 3D-printed microrobots are biocompatible. This is confirmed by incubating the particles with murine macrophage cells. As a future work, various 3D-printed biocompatible medical microrobots with various medical functions will be fabricated toward specific in vivo clinical applications.

J774A cells were allowed to reach 80% confluence, observed by optical microscopy. The cellular removal procedure is done by rinsing with dulbecco’s phosphate-buffered saline (DPBS) without Ca+ and Mg+ for 5 min. After aspiration of DPBS, a fresh addition of Dulbecco’s Essential Medium (DMEM) is added. The cells were then removed from the flask by cell scraper and counted by hemocytometer. A cell suspension of 5X103 cells per mL is created. The samples were placed within 96-well cell culture plates. After mixing, the cell suspension is added to the wells of the plate containing samples at 5K per well. After a 1 or 24 h exposure time, the plates were removed and observed by microscopy. A serial dilution of the FePt into concentrations up to 500 μg mL^1^ is added by 100 μL^1^ into the wells containing cells. A subsequent 24 h is allowed to pass, and the samples were treated with WST-8, and then, after 2h, the measurement of WST-8 is performed by spectrophotometer at 450 nm. For the different concentrations, a live/dead analysis is performed. This is performed by the addition of live/dead cell imaging kit (Thermo Fisher). The cells are incubated for 30 min, and then observed by fluorescent microscopy (inverted microscope from Nikon Instruments Inc). The fluorescent images of live and dead are collected at the emission and excitation wavelengths of 498/515 and 570/602 nm.

## Experimental Section

**Figure 1:**
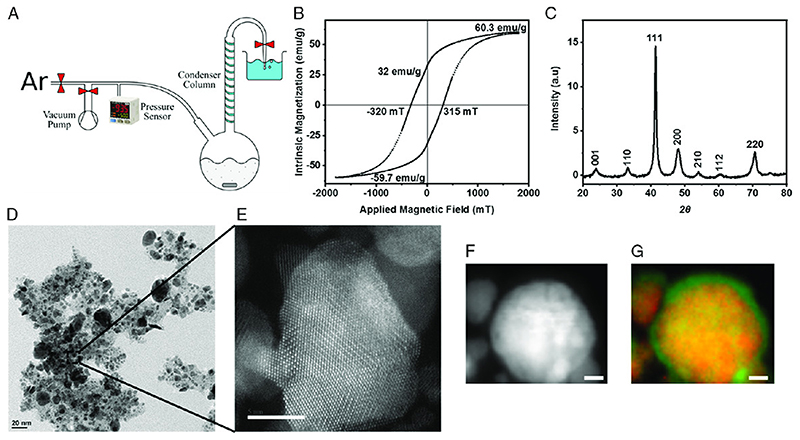
Synthesis and characterization of the iron platinum (FePt) nanoparticles for 3D-printable and biocompatible magnetic microrobots. A) The synthesis of FePt nanoparticles can be accomplished through a single-pot chemical reaction. The core components, illustrated here from left to right, are the argon source, vacuum pump, pressure sensor, round bottom flask, condenser column, and water bubbler. B) The hysteresis loop characterization of the synthesized FePt nanoparticles using VSM. The key magnetic properties are a 60emu g^-1^ saturation magnetization, a 32 emu g^-1^ remanent magnetization, and a coercive field of320 mT. C) XRD plot indicating the presence of crystalline FePt nanoparticles. D) TEM image of the FePt nanoparticles image, showing the presence of polydispersity in FePt nanoparticles. E) Magnified image of TEM showing the presence of highly crystallized FePt nanoparticle. Scale bar is 2 nm. F) EELS image of FePt nanoparticles. G) EELS color-coded image of the particles with Fe in green and Pt in red. Scale bars are 5 nm.

The synthesis procedure is derived from the literature and modified for a conventional fume hood setup.[^16a,b,21,31^] Figure 1A shows an illustration of the experimental setup, which is placed in a conventional fume hood. An argon source and vacuum pump are connected through valves to the input of a 100 mL round bottom flask. A pressure sensor (Panasonic DP-111A-E-P) is attached to monitor the absolute pressure of the system. The round bottom flask contained one side inlet and one top outlet. Before the flask is fixed with clamps and placed in a 100 mL round heating mantle (Carl Roth HCK6.1), a thermocouple is placed on the bottom of the mantle, and the flask held the thermocouple in place. Thus, the temperature between the mantle and the flask is used as a human in the loop feedback mechanism. The outlet of the flask is attached to a chilled water condenser, which initially has no water flowing through the condenser. The condenser outlet leads to a valve between the system and a pipette tip, which is inserted into a beaker of water to act as a water bubbler. An important note is that the actual length of tubing between the condenser and bubbler is approximately half a meter to prevent aspiration of water into the condenser *column.*

All chemicals were obtained from Sigma-Aldrich and used as delivered. The flask is cleaned, dried, and prepared with 20 mL of dioctylether, 388 mg, 1.5 mmol, of 1,2-hexadecanediol, 197 mg, 0.5 mmol, of platinum acetylacetonate, and a magnetic stir bar. The stirrer is kept at 800 revolution per minute (rpm) during the entire synthesis. Twice in the synthesis, an argon purge is performed. When this is done, the bubbler valve is closed, and the system is vacuumed to 5-10 kPa and filled with Argon. When pumping, the argon source is closed, and when filling, the system is over-pressured by ≈10 kPa, to an absolute pressure of ≈111 kPa. This is repeated five times. The solution is brought to 50°C, and an argon purge is performed. When the solution reached 100°C, the temperature is held for 20 min. The flask is then separated from the condenser column, and 314 mg of iron acetylacetonate, 160 μL, 0.5 mmol, of oleic acid, and 170 μL, 0.5 mmol, of oleyl amine are added to the solution. The condenser is reattached, the thermocouple is checked to still be in the center of the mantle, and an argon purge is performed. The temperature is then increased at a rate of ≈5 °C min^-1^ to 200°C, when the cooling water is turned on. The increase continues to 295-300°C, and bubbling of the solution is confirmed. The solution is then held at reflux for 30 min. The mantle heater is turned off for 1 h, and the flask is lifted out of the mantle and allowed to cool to room temperature. The solution is split into two 50 mL Falcon tubes, and 20 mL of ethanol is added to each to precipitate the particles. The solution is placed in a vortex mixer for 5 min, and the solution is centrifuged for 1 h, and the unreacted reactants are removed.

Five iterations of the cleaning step are done. Each iteration is performed as follows: First, 15 mL of hexane is added to each tube with 25 μL of oleic acid and 25 μL of oleyl amine. This is placed in a vortex mixer for 5 min. To precipitate the particles, 20 mL of ethanol is added to each tube and vortexed for 5 min. The solution is centrifuged for 20 min, and the liquid component is removed. After cleaning, the particles were concentrated into 10 mL of hexane and placed in a ceramic boat and set aside to evaporate the hexane, ≈30 min. The boat is then placed into an annealing furnace, which is evacuated and filled with forming gas, 5% H_2_ and 95% Ar. As previously mentioned, the annealing step changes the crystal structure from the FCC to FCT structure. While in the FCC phase, the material will remain magnetically soft, with small magnetic remanence and low coercivity, and in the ordered FCT phase, the particles exhibit a high magnetic anisotropy constant, leading to high coercive fields and remanent magnetization. [^15b^] The temperature is then increased by 10°Crnin^-1^ to 650°C, and held at this temperature for 1 h before being allowed to cool as rapidly as the furnace would allow before withdrawing the furnace and removing the material. The Curie temperature of annealed FePt nanoparticle systems is noted by Rong et al. to be around 600°C for a 1:1 Fe:Pt ratio and an FCT structure, so the material is guaranteed to be demagnetized after annealing.[^32^] The material is then physically gathered and stored under argon prior to use. Nickel-coated microswimmers were fabricated using IP-S photoresist (Nanoscribe GmbH). The 3D-printed microswimmers were sputtered with 100 nm Ni and 50 nm Au film using a benchtop sputter coating system (Leica EM ACE600, Leica Microsystems); 5mgmL^-1^ polyethylene glycol (PEG)-amine-coated 50 nm iron oxide nanoparticles were dispersed into PETA solution with 3% (w/v) Irgacure 369 (fluidMAG-PEG/Amine, Chemicell GmbH). The pre-polymer solution is sonicated for 2 h to get complete dispersion.

Murine macrophage cells (J774A) were purchased from ATCC. The cells were characterized by surface markers (CD11b, CD80, CD206), morphology, and ability to phagocyte. The cell culture medium is prepared as DMEM (Gibco) supplemented with 10% heat-inactivated Fetal Bovine Serum (HI-FBS) (Gibco) with 1% Penicillin Streptomycin (Gibco). The cells were thawed from cryopreservation at passage 3. The cell culture is all performed in a biosafety cabinet. All cells were stored under cell culture conditions, 5% CO_2_, 80% humidity, and 37°C. The cellular passages for the experiments are between passage 5 up to passage 25.

## Supplementary Material

Supplementary data

Supplementary video 1

Supplementary video 2

## Figures and Tables

**Figure 2:**
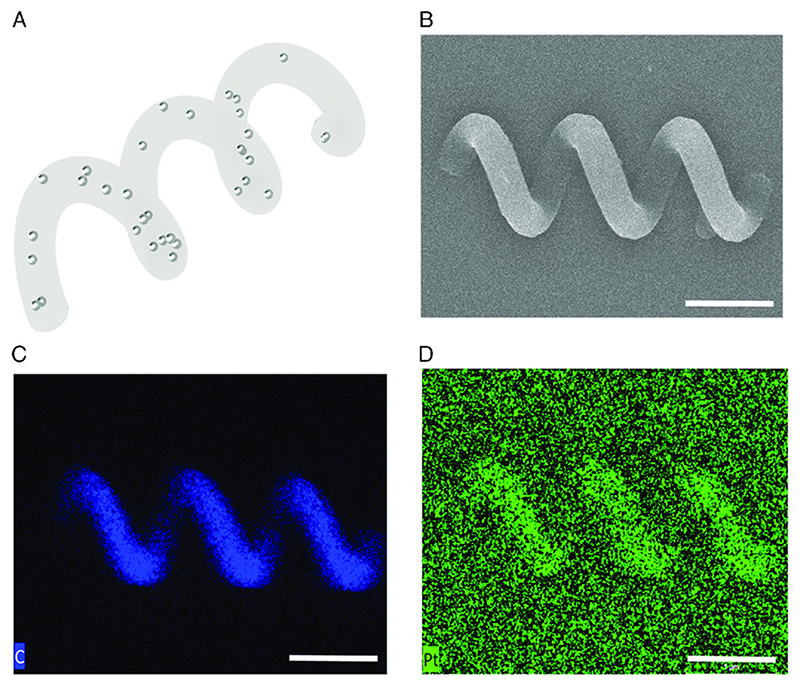
3D-printed magnetic helical microswimmer prototype using two-photon polymerization. A) Concept illustration, where a PETA magnetic helix with three turns has embedded FePt nanoparticles distributed throughout its body. B) SEM image of a printed helix. C) EDX images of the magnetic helix. C) The strong carbon signal from the PETA part shows the location of the spiral. D) The Pt signal in the EDX image (blue color), while weaker than the carbon signal, visibly shows the same location of the spiral, demonstrating that the FePt particles are evenly distributed throughout the helical structure. Scale bars represent 10 μm.

**Figure 3:**
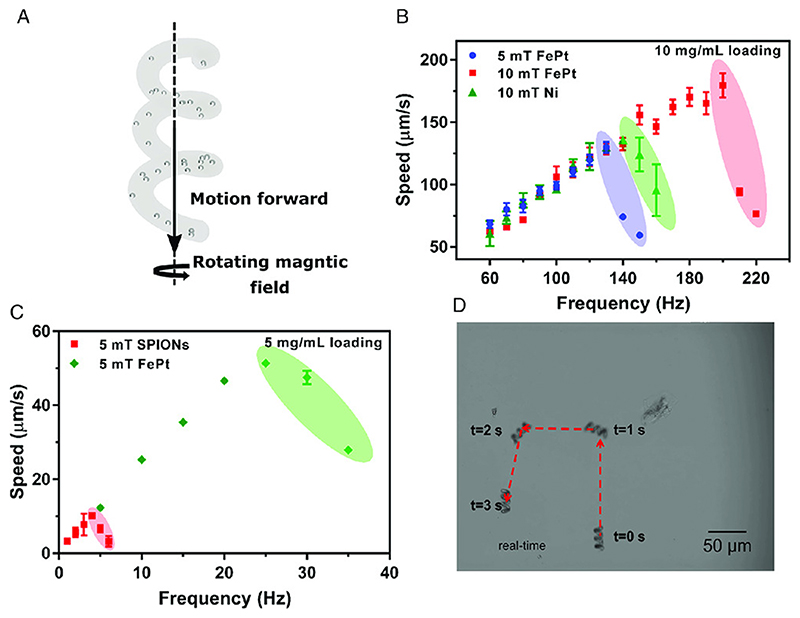
Swimming mobility characterization of the printed PETA helical microswimmers with embedded FePt nanoparticles. A) Concept illustration of a PETA microswimmer with embedded FePt nanoparticles showing the velocity vectors of the microswimmers with forward and rolling velocity components. B,C) Swimming performance of the 30 μm long FePt/PETA and SPION/PETA magnetic helices with speeds as a function of the rotating magnetic field frequency at a given magnetic field magnitude. B) 5 mg mL^1^ loading of FePt is compared with 5 mg mL^1^ loading of SPIONs. A performance increase of approximately a factor of fivefold increase is achieved. C) Speeds of 180 μm s^1^, six body lengths per second, are achieved at a step out frequency of 200 Hz for a 10 mT rotating field, higher than a 100 nm thick Ni-coated helical swimmer. D) Example trajectory of a microswimmer actuated by a rotating magnetic field with a 10 mT magnitude and 50 Hz frequency. Scale bar is 50 μm.

**Figure 4:**
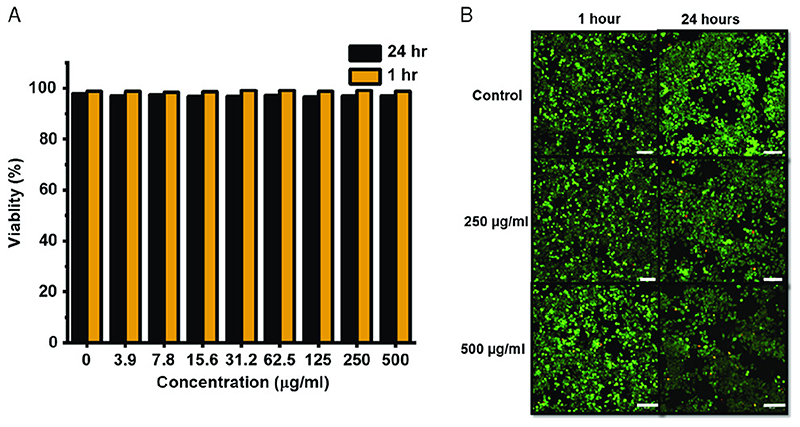
In vitro J774A cell viability tests in the presence of FePt nanoparticles. A) Cell viability as a function of FePt nanoparticle concentration after 1 and 24 h of incubation in the presence of the particles. B) Optical fluorescent images of the J774A cell samples from the control, 250 and 500 pgmL^-1^ showing inhibited cell division in the higher concentration sample after 24 h. The green and red colors indicate live and dead cells. Scale bar is 100 μm.

**Table 1:** Properties of the common magnetic materials as particles or films used in the fabrication of magnetic microrobots. 1 emu is equivalent to 10^-3^ Am^2^.

Material	Saturation magnetization	Remanent magnetization	Coercive field [mT]
FePt nanoparticles (this article)	60 emu g^-1^	32 emu g^-1^	320
FePt nanoparticles (Kadiri et al.)^[21]^	0.7 emu mm“^-3^	0.65 emu mm^-3^	1500
FePt nanoparticles (Shima et al.)[^16c^]	1 emu mm^-1^	1 emu mm^-1^	3600
NdFeB microparticles[^26^]	1.1 emu mm"^3^	0.73 emu mm"^3^	700
Cobalt film^[27]^	0.76 emu mm^-3^	0.2 emu mm^-3^	22
Nickel film	55.1 emug^-1^[^28^]	0.28-0.50 emu mm^-3^[^29^]	0.2-29[^24^,^29^]
SPIONs^[30]^	45 emu g^-1^	0	0
