# Supplementary material for: 3D Microprinting of Iron Platinum Nanoparticle-Based Magnetic Mobile Microrobots
Source: Adv Intell Syst. Author manuscript; Available in PMC 2021 Mar 29. (PMC7610460; doi:10.1002/aisy.202000204)
Supplement: Supplementary data [file EMS119907-supplement-Supplementary_data.pdf]

Supporting Information

3D-Microprinting of Iron Platinum Nanoparticle-based Magnetic Mobile Microrobots

Joshua Giltinan[1,*], Varun Sridhar[1,*], Ugur Bozuyuk[1], Devin Sheehan[1], and Metin Sitti[1,2,3,†]

[1] Physical Intelligence Department, Max Planck Institute for Intelligent Systems, Stuttgart, 70569, Germany

[2] School of Medicine and School of Engineering, Koç University, 34450 Istanbul,Turkey

[3] Institute for Biomedical Engineering, ETH Zurich, 8092 Zurich, Switzerland

[*] Equally contributing first authors

[†] Correspondence to: sitti@is.mpg.de

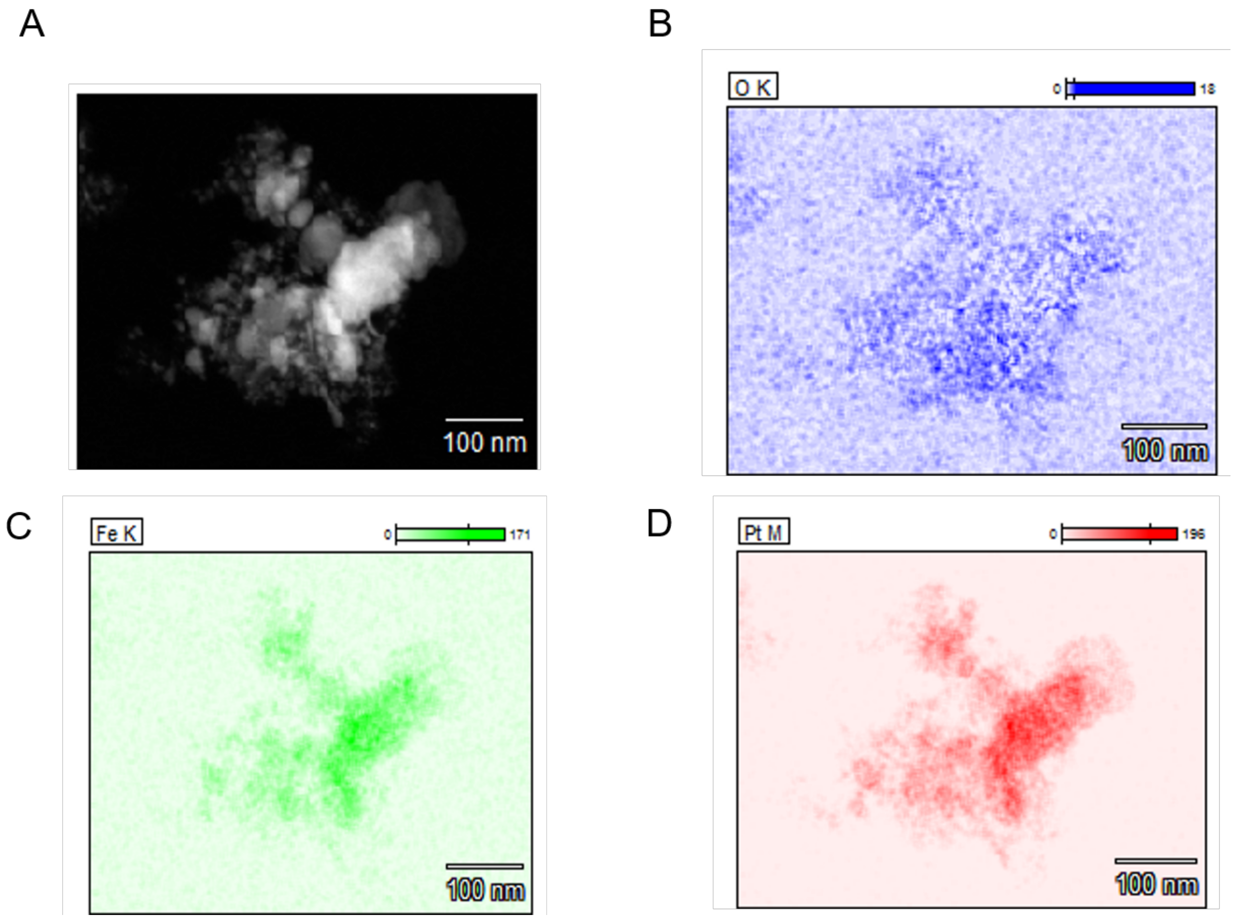

Figure S1. Energy dispersive X-ray (EDX) spectroscopy images from TEM of FePt nanoparticles. A) Bright field image of FePt nanoparticles. B-D) elemental images of O, Fe and Pt in the FePt nanoparticles indicating the composition to be 58% Fe and 42% Pt.

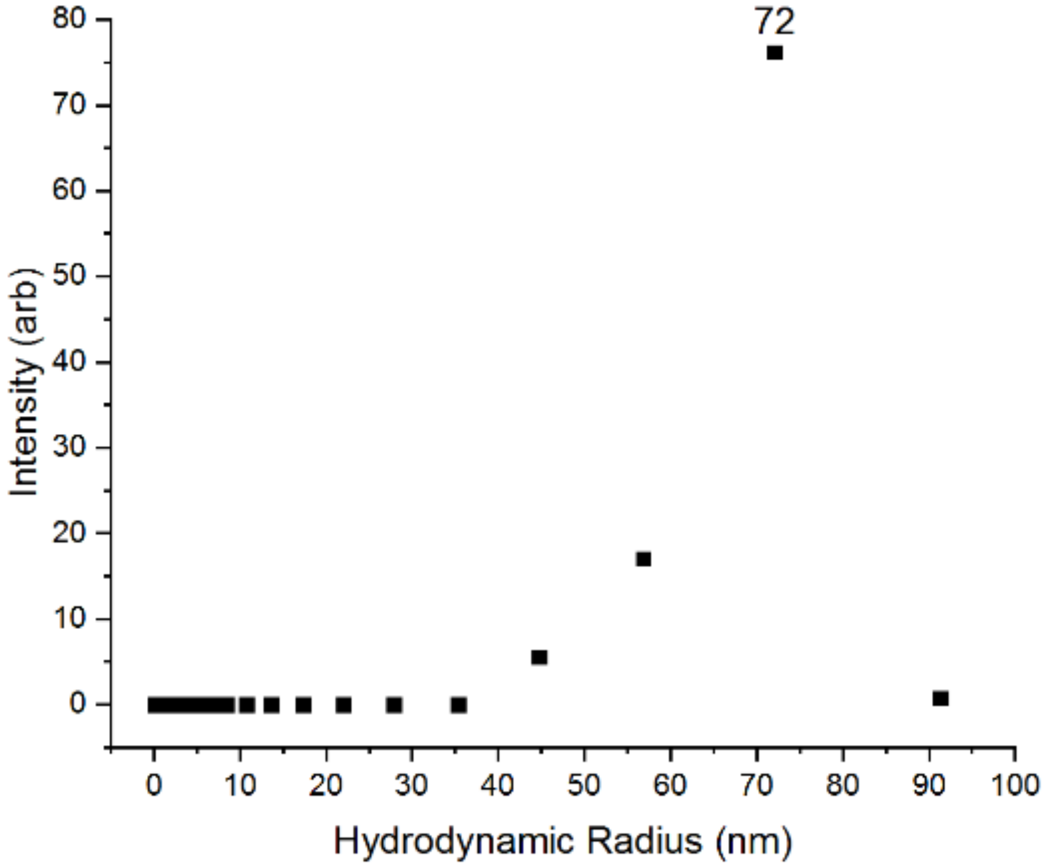

Figure S2. Dynamic light scattering (DLS) analysis of the synthesized FePt naoparticles indicating the hydrodynamic radius.

Supplementary Movies

Supplementary Movie 1: Magnetic actuation of the FePt nanoparticles embedded helical microswimmer at 10mT and 50 Hz near the surface.

Supplementary Movie 2: Magnetic out of plane, away from the surface actuation of the FePt nanoparticles embedded helical microswimmer at 10mT and 50 Hz.
